# The case for DUF1220 domain dosage as a primary contributor to anthropoid brain expansion

**DOI:** 10.3389/fnhum.2014.00427

**Published:** 2014-06-24

**Authors:** Jonathon G. Keeney, Laura Dumas, James M. Sikela

**Affiliations:** Department of Biochemistry and Molecular Genetics and Human Medical Genetics and Neuroscience Programs, University of Colorado School of Medicine, Anschutz Medical CampusAurora, CO, USA

**Keywords:** DUF1220, protein domain, copy number, human brain expansion, gene duplication and evolution, gene dosage, anthropoid brain evolution

## Abstract

Here we present the hypothesis that increasing copy number (dosage) of sequences encoding DUF1220 protein domains is a major contributor to the evolutionary increase in brain size, neuron number, and cognitive capacity that is associated with the primate order. We further propose that this relationship is restricted to the anthropoid sub-order of primates, with DUF1220 copy number markedly increasing in monkeys, further in apes, and most extremely in humans where the greatest number of copies (~272 haploid copies) is found. We show that this increase closely parallels the increase in brain size and neuron number that has occurred among anthropoid primate species. We also provide evidence linking DUF1220 copy number to brain size within the human species, both in normal populations and in individuals associated with brain size pathologies (1q21-associated microcephaly and macrocephaly). While we believe these and other findings presented here strongly suggest increase in DUF1220 copy number is a key contributor to anthropoid brain expansion, the data currently available rely largely on correlative measures that, though considerable, do not yet provide direct evidence for a causal connection. Nevertheless, we believe the evidence presented is sufficient to provide the basis for a testable model which proposes that DUF1220 protein domain dosage increase is a main contributor to the increase in brain size and neuron number found among the anthropoid primate species and that is at its most extreme in human.

## Hypothesis: anthropoid primate brain expansion is primarily driven by DUF1220 domain dosage

Here we provide an overview of the data supporting the view that DUF1220 copy number (dosage) increase is a major contributor to the increase in neuron number, brain size, and cognitive ability that has occurred specifically in the anthropoid sub-order (monkeys, apes, humans) of primates, including the evolutionarily rapid and extreme expansion of the human brain.

### Expansion of the primate brain has occurred via an evolutionary mechanism unique to primates

Recent studies have indicated that, among primates, brain expansion has been driven by unique neuronal scaling rules such that larger brains can be accounted for primarily by gains in neuron number, unlike any other lineage so far investigated (Herculano-Houzel et al., 2007). Although this trend has not yet been investigated in lineages that contain large brained animals such as pachyderms and cetaceans, this phenomenon may help to explain differences between the cognitive abilities of humans and other large brained animals, such as elephants, whales, and dolphins. This is a significant finding that has a number of important implications. First, it indicates that cell size in the mammalian brain is not a fixed value, and secondly that neuron number across species must therefore be evaluated independently of brain size.

As has recently been reported, neuronal scaling, and density appear to vary by taxonomic order, with primate brain evolution taking its own unique path (Herculano-Houzel, 2009). It has been pointed out that body size might not be a relevant parameter for defining a species' behavioral performance. Rather, absolute numbers of brain neurons might be a much more important factor. This idea is based on the finding that a larger body size between two rodents of different species seems to be accompanied by an increase in brain size due in large part to an increase in neuron size, and therefore a reduction in neuron density (Herculano-Houzel et al., 2006). In primates, on the other hand, neuron size and density stay nearly the same. Cell number therefore accounts for a much larger proportion of the difference in brain mass between primates as compared to the differences in brain mass between rodent brains. This shift in emphasis from cell mass to cell number seems to be unique to the primates as an order, rather than humans alone, suggesting that primate brain expansion has proceeded by a different mechanism than that used by non-primates.

Mechanistically, this model also makes intuitive sense. Because neurons are fundamentally on/off switches, there is no intrinsic advantage for an animal to have larger neurons. Indeed, the extra energy required to maintain larger cells in a larger animal that are performing the same function may instead be a disadvantage, offset only by the advantage provided by a larger overall body size.

By contrast, there is a distinct advantage in processing power in an animal that is able to keep neuron size to a minimum and instead increase neuron number. Additionally, neurons can be considered the fundamental units of cognitive power. Having more neurons likely produces higher cognitive function by expanding the processing capacity of input, processing, and association regions. This should in theory allow an animal to have added layers of pattern recognition and connect increasingly more complex outputs, and therefore be capable of producing more complex thought patterns. Because primates tend to have more neurons than non-primates of equal brain size, this provides a plausible explanation for the observed greater cognitive capacity of primates when compared to non-primate mammals.

Taken together these findings support the view that among mammalian species, brain evolution in primates has occurred by a different mechanism. Namely, that the increase in brain size and cognitive ability found among primates, and particularly humans, has been primarily due to an increase in neuron number.

### DUF1220 protein domain copy number increase shows a primate-specific burst that closely parallels brain size and neuron number among primate species

#### The highest number of DUF1220 copies is found in the human lineage

It has been well-established that extreme gene duplication in a species-specific manner can be a key contributor to the evolution of phenotypic traits unique to that species. In order to identify such extreme duplication events, we previously used genome-wide and gene-based arrayCGH to identify lineage-specific gene copy number changes among primate species including human (Fortna et al., 2004). This study surveyed essentially all human genes (e.g., approximately 24,000) and identified 1,004 that showed lineage-specific copy number changes among humans and the 4 great ape species. Of 140 genes identified that showed human lineage-specific (HLS) gain or loss in copy number, the most extreme change was found to be due to sequences encoding DUF1220, a protein domain of unknown function (Popesco et al., 2006). DUF1220 domains are on average approximately 65 amino acids in size, are found almost exclusively within the *NBPF* gene family (Vandepoele et al., 2005) and are located primarily in the 1q21.1 region of chromosome 1.

There are many more copies of DUF1220 encoded in the human genome compared to the genome of any other species (Table 1). Humans have approximately 270 haploid copies, far more than great apes (90–125 copies), monkeys (25–40 copies), and especially prosimians and non-primate mammals (1–9 copies) (O'Bleness et al., 2012a). Indeed, DUF1220 shows the largest HLS increase in copy number of any protein coding sequence in the human genome (O'Bleness et al., 2012b).

**Table 1:** **DUF1220 Copy Number in 41 Animal Genomes**.

	**Genome**	**PDE4DIP**	**Total DUF1220**	**NBPF Genes**
**EUARCHOTANGLIRES**				
Anthropoids	Human	2	272	23
	Chimp	3	125	19
	Gorilla	3	99	15
	Orangutan	4	92	11
	Gibbon	3	53	10
	Macaque	1	35	10
	Marmoset	1	31	11
Prosimians	Mouse Lemur	1	2	1
	Bushbaby	1	3	2
	Tarsier	1	1	0
	Rabbit	1	8	3
	Pika	1	1	0
	Mouse	1	1	0
	Rat	1	1	0
	Guinea Pig	1	1	1
	Squirrel	1	1	1
	Tree Shrew	1	4	3
**LAURASIATHERIA**				
	Cow	1	7	3
	Dolphin	1	4	1
	Pig	1	3	1
	Horse	1	8	3
	Dog	1	3	1
	Panda	1	2	1
	Cat	1	3	2
	Megabat	1	1	0
	Microbat	1	1	0
	Hedgehog	1	1	0
	Shrew	1	1	0
**XENARTHRA**				
	Armadillo	1	1	0
	Sloth	1	1	0
**AFROTHERIA**				
	Elephant	1	1	2
	Hyrax	1	1	0
	Tenrec	1	1	0
**METATHERIA**				
	Opossum	1	1	0
	Wallaby	1	1	0
**PROTOTHERIA**				
	Platypus	1	1	0
**OTHER VERTEBRATE**				
	Chicken	0	0	0
	Zebra Finch	0	0	0
	Lizard	0	0	0
	Frog	0	0	0
	Zebrafish	0	0	0

DUF1220 copy number was estimated using BLAT searches of 41 available animal genomes as described in O'Bleness et al. (2012a). PDE4DIP refers to the copy number of the ancestral form of DUF1220, which is found only in the PDE4DIP gene. Also listed is the copy number of the NBPF genes, i.e., those containing the derived form of DUF1220 and that expanded particularly in the anthropoid species.

It is worth noting that these increases occur in the DNA sequences of a gene coding region and specifically in a protein domain, characteristics that make them much more likely to be functionally and phenotypically important. Their potential significance is further supported by molecular evolutionary analyses of DUF1220 coding sequences which show that DUF1220 domains have undergone strong positive selection especially among primates (Popesco et al., 2006).

#### DUF1220 increase in humans is primarily due to hyper-amplification of the DUF1220 triplet

We have estimated that approximately 160 copies of DUF1220 were added specifically to the human genome since the *Homo/Pan* lineages diverged from one another approximately 5–6 million years ago. This extraordinary expansion corresponds to an average of ~28 copies added specifically to the human genome every million years since this split. However, phylogenetic analyses of the new HLS copies suggests that this expansion did not follow a constant rate of increase, and particularly large bursts of DUF1220 copy number likely occurred in very recent human evolution (e.g., within the last 1–2 million years) (O'Bleness et al., 2012a).

It is noteworthy that the most extreme HLS increase in DUF1220 copy number involves intra-genic domain amplification, rather than gene duplication. DUF1220 protein domains can be divided into six clades based on sequence similarity: Conserved clades 1, 2, and 3 (CON1-3) and Human Lineage Specific clades 1, 2, and 3 (HLS1-3). The clades exhibit specific positions within *NBPF* genes, with CON1, and CON2 being the N-terminal-most domains followed by the three HLS clades with CON3 being the C-terminal-most domain (O'Bleness et al., 2012a). Interestingly, the three HLS DUF1220 clades combine to make up a repeating unit comprised of three HLS DUF1220 domains (HLS1, HLS2, HLS3) that we have named the HLS DUF1220 triplet. The HLS expansion of this triplet represents one of the most extreme, rapid, and recent copy number expansions found in the human genome (O'Bleness et al., 2012b). We estimate that remarkably 148 of the 160 DUF1220 copies added specifically to the human lineage since the *Homo/Pan* split were due to intragenic hyper-amplification of the HLS DUF1220 triplet.

A phylogenetic profile of DUF1220 expansion in the human lineage shows that, because the triplets are so similar, it is likely the majority were generated very recently in human evolution, perhaps within the past 500,000 years (O'Bleness et al., 2012a). More precise estimation of when the DUF1220 triplet hyper-amplification occurred should be obtained as more complete and accurate genome sequences become available from evolutionarily recent human relatives, e.g., Neanderthals, Denisovans.

Finally, the data presented strongly suggests that this exceptional DUF1220 copy number increase has conferred a considerable evolutionary advantage to anthropoid primates, first among monkeys, then apes, and most recently and extremely among humans.

#### DUF1220 copy number shows a strong correlation with brain size, neuron number, and other cognitive phenotypes among primates

When compared across primates, DUF1220 copy number shows a remarkably close correlation with cortical neuron number (*R*^2^ = 0.98; p = 1.1 × 10^−3^) and brain size (*R*^2^ = 0.98; p = 1.8 × 10^−6^) (Dumas et al., 2012). Further examination of brain measurements in 131 individuals from 7 Catarrhini species (Old world monkeys, apes, and humans), made using MRI, reveals significant correlations between DUF1220 domain dosage and increases in gray matter (*p* = 1.3 × 10^−4^), surface area (*p* = 7.8 × 10^−5^), gyrification (*p* = 2.0 × 10^−3^) and white matter (*p* = 2.1 × 10^−5^) (Keeney et al., in press). However, no significant correlation is found when DUF1220 copy number is compared to primate body size.

While there has been an unusually large and rapid increase in brain size specifically in humans, this is part of a process that has a long evolutionary history. Several neurobiological trends, such as an expanded neocortex in humans, are an extension of an evolutionary direction already begun in the brains of other primates that was evident well before the human lineage diverged from the *Pan* lineage (chimp and bonobo). It is estimated that 30–40 Mya neocortical portions of the brain increased in the two emerging anthropoid lineages (platyrrhines and catarrhines) and 8–16 Mya another enlargement occurred in the lineage to the modern hominids (Goodman, 1999; Sikela, 2006). Still, the largest neocortical increase is thought to have occurred over the past 3 million years in the human lineage.

This continued progressive increase in primate brain size over 60 million of years of evolution shows a strikingly close parallel with the sustained and stepwise copy number expansion of DUF1220 domains among these species. As mentioned above, the most extreme DUF1220 copy number expansions are those that are restricted to the human species and are likely to have occurred via small and large incremental increases. Thus, while the strategy of producing more neurons to facilitate brain expansion is restricted to the anthropoid primate sub-order, so too is the extraordinary evolutionary burst in DUF1220 copy number. While these findings are consistent with DUF1220 dosage playing a significant role in anthropoid brain expansion, the supportive data are at present correlative. Additional studies are underway that more directly test the involvement of DUF1220 dosage in brain size and neuron number (see Author Note).

#### DUF1220 protein domain copy number in neanderthals

It has been long established that Neanderthals are thought to have had, on average, a larger brain than modern humans (Holloway, 1985) and recent genome sequence analysis of Neanderthals indicates that they also have more DUF1220 copies (~350) than modern humans (~270) (Dumas et al., 2012; O'Bleness et al., 2012a). This lends further support to the view that DUF1220 dosage is associated with brain size increase among all primate species studied, including humans and Neanderthals.

However, the fact that Neanderthals show the greatest number of DUF1220 copies among all mammals raises a number of questions: First, because of the imprecise nature of the available Neanderthal sequence we do not currently know which types of DUF1220 copies are found in the Neanderthal genome. As mentioned above (section DUF1220 Increase in Humans is Primarily due to Hyper-Amplification of the DUF1220 Triplet), in humans DUF1220 domains are found in six similar clades that are organized in a specific order within the *NBPF* genes that encode them. While some clades have been more strongly implicated in brain-size related phenotypes, the exact functions of the different clades are not known. Secondly, we do not know the cause of the Neanderthal extinction, and therefore we cannot make conclusions about whether brain function and/or cognitive capacity played a role in this process. There are several theories that propose different types of causes for the Neanderthal extinction (Golovanova et al., 2010), and many of these do not necessarily involve changes in brain function and/or ability. Finally, while recent studies indicate that Neanderthals were not as brutish as once suspected (Rendu et al., 2013) many questions still remain regarding the level of Neanderthal cognitive ability. In recent years there has been an increasing number of reports that portray Neanderthals as more culturally developed than had been previously thought (Soressi et al., 2013).

In summary, given our limited knowledge in the above areas, we cannot at present make conclusions regarding whether the increased number of DUF1220 copies in Neanderthal conferred any evolutionary advantage or disadvantage to that subspecies. Nevertheless, the correlation between DUF1220 copy number and brain size is retained even in this close human relative.

#### The evolutionary advantage of brain evolution being driven by dosage increase

Of the potential genomic processes that could have produced the evolutionarily rapid increase in neuron number among primates, a mechanism involving copy number (dosage) increase would offer a number of theoretical advantages. For example, if more of a gene product conferred an evolutionary advantage, further increasing copy number (dosage) would allow an increasingly greater amount of product to be easily generated, presumably delivering increasingly greater evolutionary benefit.

From this perspective, by relying on DUF1220 dosage increase as the primary means of expanding neuron number, brain size, and cognitive capacity, the primate order has fortuitously utilized a unique mechanism that is highly expandable, i.e., it allows continued improvements to be generated simply by duplicating more and more DUF1220 domains, i.e., increasing DUF1220 domain dosage (Dumas and Sikela, 2009). In this scenario, evolutionary variation in the DUF1220 sequence may not be as critical as variation in copy number. Also, it is noteworthy that a recent study has reported that DUF1220 (i.e., the *NBPF* protein product) may encoding a transcription factor (Zhou et al., 2013). If correct, this finding suggests a plausible means by which an increase in dosage could have a dramatic functional impact.

So why do other mammals with larger brains (elephants, whales, e.g.,) not show the same progressively higher cognitive capabilities as found in primates and particularly in humans? The large increases in DUF1220 copy number are found only among anthropoid primate species, and thus it is plausible that evolutionary brain expansions in non-primate species have occurred using genomic mechanisms other than the DUF1220 copy number increase utilized by primates (see Author Note). In the event this is true, it could explain why the brains of these other “intelligent” species have not approached or surpassed the cognitive abilities of the human brain. In other words, non-primate brain evolution did not use the same easily “expandable” genomic mechanism for brain size increase, and instead used ones that do not lend themselves to frequent and recurrent expansions in brain size and cognition. Indeed, it has been estimated that the types of large scale recombination events that would produce copy number variations (CNVs) occur roughly 2–3 orders of magnitude more frequently than the types of point mutations that might affect an alternative mechanism of gene regulation (Itsara et al., 2010).

It should also be noted that a prediction of the above argument is that, when measurements of neuron number become available for large brained non-primates (elephants, cetaceans), the neuron numbers will not be as extreme as those found in anthropoid primates (and particularly human). In summary, increasing cognitive capacity through increases in gene or domain copy number provides a much more open-ended mechanism, allowing more and more cognitive enhancement by the relatively simple genomic process of adding more and more copies (i.e., increasing dosage) of the critical DNA sequences.

### DUF1220 evolution and genome organization

#### Human DUF1220 sequences are located in an evolutionarily dynamic genomic region

The great majority of human DUF1220 sequences are located in 1q21, one of the most evolutionarily dynamic regions of the genome (Fortna et al., 2004; Popesco et al., 2006; O'Bleness et al., 2012a). This region is adjacent to the human-specific C-band at 1q12. C-bands have long been recognized as cytogenetically visible chromosomal landmarks that are largely composed of heterochromatin and are polymorphic in the human population. Three other human specific C-bands are found at 9q12, 16 and Y, and these along with the one at 1q21.1 are adjacent to clusters of genes that exhibit HLS increases in copy number in the genome (Fortna et al., 2004; O'Bleness et al., 2012b). Interestingly, the 1q21 region and flanking 1q12 region are positioned within a HLS pericentromeric inversion that contains almost all of the 160 DUF1220 copies that were added specifically to the human lineage since our split with the *Pan* lineages.

#### Selection has focused primarily on increasing the amount of DUF1220 protein

DUF1220 is a protein domain of approximately 65 amino acids and molecular analysis of DUF1220 coding sequences indicates that DUF1220 domains show strong signatures of positive selection particularly among primates (Popesco et al., 2006). But it is also clear that, among anthropoid species and particularly in humans, evolution has primarily selected for more DUF1220 copies, many of which are virtually identical, rather than for a highly diverged family.

This selection pressure on coding sequences notwithstanding, it appears from the unprecedented increase in human copy number and the limited sequence divergence among human DUF1220 copies, that selection has primarily favored increasing the amount of DUF1220 protein that can be produced (though such quantifications are difficult to assess in practice, as they likely require fetal brain tissue from each species). The importance of dosage increase is further borne out by the observation that the HLS DUF1220 triplet, which by adding approximately 148 DUF1220 copies to the human genome essentially doubled the number of human DUF1220 copies, shows remarkably high sequence conservation (O'Bleness et al., 2012a). This suggests that in anthropoid species more copies of DUF1220 produce a greater dosage of the DUF1220 protein domain, and that these products are acting to enhance or amplify a particular function. One such plausible function would be to increase neuron number, which we have previously mentioned closely parallels DUF1220 copy number among primates.

Recent data has provided further evidence that selection pressures have focused primarily on increasing DUF1220 dosage. The number of DUF1220 domains encoded by each of the 20 human *NBPF* genes covers a broad range (from 5 to 67 copies) (O'Bleness et al., 2012a). Surprisingly, rather than many different sized proteins being produced (as would be expected from the DUF1220 domain distribution in the *NBPF* gene family) only a primary DUF1220 domain-including protein of 37 kDa is produced when evaluated by Western Blot (Popesco et al., 2006). In theory, this product could accommodate 3–4 DUF1220 domains, though its composition is still under investigation. Nevertheless, this discovery suggests that this size-restricted protein product may represent the major functional form of DUF1220 in human brain and other tissues.

#### DUF1220 sequences are one of several core duplicons in the human genome

A systematic genome-wide study showed that a select group of sequences have undergone unusually rapid copy number expansions in the human genome and, in so doing, have “dragged” different flanking sequences along with them as they duplicated and moved to new locations (Marques-Bonet and Eichler, 2009). These sequences have been termed “core duplicons” and DUF1220 domains are the main core duplicon on chromosome 1. While the DUF1220 copy number change fits with it being a core duplicon, so does the location of DUF1220 sequences: they are clustered but are inserted both in a tandem and an interspersed manner. While such a configuration promotes genomic variability and rapid evolutionary change, it also produces a highly disease-prone genomic architecture (as described further in the next section).

#### DUF1220 increase in humans has been accompanied by the creation of a large disease burden

The greatest increase in DUF1220 copy number in humans has occurred in the 1q21 region where approximately 240 of the 270 human DUF1220 sequences are located. The architecture of this region is characterized by the presence of several *NPBF* (DUF1220-encoding) genes that are typically interspersed by one or more low copy number non-NBPF genes. In addition, each *NBPF* gene encodes 5–60 DUF1220 domains tandemly arranged on each gene. Thus, large numbers of DUF1220 copies are found in this region in both interspersed and tandem arrangements. Such an organization is an ideal genomic environment for recombination events (e.g., NAHR) to occur, and these will often involve low-copy, dosage-sensitive genes that flank *NBPF* genes that undergo gains or losses in copy number due to the action of DUF1220 core duplicons.

This prediction has been borne out by the discovery of a large number of 1q21-CNVs that are disease-associated. At current estimate, there are at least one dozen diseases that have been reported that are associated with 1q21 CNVs, most of which are immediately flanked by or encompass DUF1220 domains (Dumas et al., 2012; O'Bleness et al., 2012b). Given the data linking DUF1220 dosage increase to evolutionary adaptation (i.e., increase in brain size and cognitive ability), we have suggested that this unusually large disease burden can be viewed as the price that our species has paid, and continues to pay, for the rapid and extreme evolutionary increase in DUF1220 copy number and for the adaptive benefit this has conferred.

In support of this idea, we have developed a model that proposes the following (Dumas and Sikela, 2009). Increased DUF1220 copy number confers an evolutionary advantage that is likely related to increases in brain size and cognitive ability. Because the 1q21 region is very unstable, this increased the likelihood DUF1220 copy number would increase. Individuals who exhibited the increased DUF1220 copy number would have a selective advantage, which in turn would result in retention of the 1q21.1 instability in these individuals. Thus, the evolutionary benefit of rapidly increasing DUF1220 copy number in the human lineage resulted in favoring retention of the high genomic instability of the 1q21.1 region. This in turn, has precipitated a spectrum of recurrent human brain and developmental disorders, including autism and schizophrenia.

Finally, DUF1220 sequences themselves are also thought to be major contributors to the current instability of the 1q21 region. Interspersed among the 53 genes in the 1q21.1 region are more than a dozen *NBPF* genes that together encode approximately 240 DUF1220 domains. Such a duplication-rich genome architecture provides the potential for numerous different recombination scenarios that could be mediated by DUF1220 sequences that would result in gain or loss of the many intervening non-*NBPF* genes in the region. Thus, given the often deleterious consequences of these types of rearrangements, it is not surprising CNVs in this region have already been linked to so many phenotypically diverse disorders (Dumas et al., 2012).

### DUF1220 dosage is linked to brain size differences within the human species

#### DUF1220 copy number and microcephaly and macrocephaly

Multiple independent reports have shown that deletions and duplications in 1q21 are associated with microcephaly and macrocephaly, respectively (Brunetti-Pierri et al., 2008; Mefford et al., 2008). These findings strongly suggest that the copy number of one or more sequences in this region is directly influencing human brain size. These CNVs, while containing a number of non-DUF1220 encoding genes, also either encompass or are immediately flanked by DUF1220 domains (Dumas and Sikela, 2009). Recently we used specialized DNA micro-arrays covering the 1q21 region for an arrayCGH analysis of 42 individuals with microcephaly or macrocephaly, that had been previously shown to contain 1q21 deletions or duplications. In this study, DUF1220 copy number exhibited the highest consistent correlation with head circumference among all 1q21 genes that were examined (*N* = 53) (Dumas et al., 2012).

Finally, of the genes in the 1q21.1 CNVs where dosage has been linked with brain size, only those encoding DUF1220 domains show such an extreme HLS evolutionary change (O'Bleness et al., 2012a) that also parallels brain size increase among anthropoid species (monkeys, apes, human).

#### DUF1220 dosage is associated with brain gray matter volume in a non-disease population

To investigate whether DUF1220 copy number influences brain size in a non-disease population, arrayCGH was carried out using DNA from 59 otherwise normal individuals for which brain MRI profiles had been obtained. Results from non-disease individuals at the two extremes of gray matter volume (as determined by MRI), indicate that the large brain group had more DUF1220 copies than the small brain group (CON1 *p* = 0.0246 and CON2, *p* = 0.0134). No other genes in the 1q21 region (*N* = 53) showed the consistent correlation between brain size and copy number found for DUF1220-related sequences among these non-disease and disease (1q21-associated microcephaly and macrocephaly) populations. Finally no genes in the 1q21 region, or for that matter anywhere else in the genome, show the extreme human copy number expansion observed for DUF1220 domains that also closely parallels increases in primate brain size (Dumas et al., 2012).

#### DUF1220 copy number, autism, and schizophrenia

It has been proposed that autism and schizophrenia may be opposite extremes of the same cognitive pathology (Crespi, 2013). This model is supported in part by studies of disease-related CNVs in which deletions are associated with one condition while reciprocal duplications are associated with the other condition. Of particular relevance here is the finding that 1q21 duplications have been associated with autism while reciprocal 1q21 deletions have been associated with schizophrenia (Crespi, 2013). As we noted earlier, these CNVs either encompass or are flanked by numerous DUF1220 domain sequences (Dumas and Sikela, 2009). In addition, individuals with autism tend to have larger than average brains or specific brain regions (reviewed in Corchesne et al., 2007), while individuals with schizophrenia tend to have smaller than normal brains (Haijma et al., 2013), suggesting that the dosage of key gene-related sequences that underlie these disorders may also play a role in influencing brain size. It terms of 1q21 genes, we have recently shown that of 53 genes that map to 1q21, DUF1220-related sequences show the most consistent correlation with brain size among both normal and disease-associated populations (Dumas et al., 2012). Such findings provide further support to the notion that DUF1220 may be linked to autism and schizophrenia.

It has also been suggested that the same key genes that have been important to human-specific brain evolution will be shown to also underlie schizophrenia (Crow, 1995), a possibility that fits with what we know about DUF1220 domains being important to human evolution. More recently we have found a more direct link between DUF1220 and ASD. Namely that, in individuals with ASD, DUF1220 subtype CON1 is linearly associated, in a dose response manner, with increased severity of each of the three primary symptoms of ASD: increased social deficits (*p* = 0.021), increased communicative impairments (*p* = 0.030), and increased repetitive behaviors (*p* = 0.047) (Davis et al., 2014). Finally, it is worth noting that DUF1220 copy number in the human population is highly polymorphic (for example, copy number of the DUF1220 CON1 subtype ranges from 56 to 88 and follows a Gaussian curve) (Davis et al., 2014). This feature implies that the DUF1220 family, by itself, provides a rich source of functional allelic variation and as such has the capacity to produce a broad continuum of phenotypic effect. While these findings support a potential role for DUF1220 in autism and schizophrenia, a more detailed discussion of this possibility is being presented elsewhere.

## Models of how DUF1220 domain dosage could be driving neuron number and brain size

The data presented above suggest a possible role for DUF1220 dosage in influencing neuron number in the primate brain. There are several plausible mechanisms through which this may be brought about and these are described in the following section.

### DUF1220 and the centrosomal effect on brain size

Virtually all genes that have been implicated in primary microcephaly encode proteins that function at the centrosome, a fact that has led to the suggestion that genes that act at the centrosome may play key roles in the regulation of brain size (Thornton and Woods, 2009). In light of this, it is noteworthy that the ancestral DUF1220 domain is encoded by a large gene, phosphodiesterase family member 4D interacting protein (PDE4DIP or myomegalin), that is also expressed at the centrosome (Verde et al., 2001). Interestingly, one of the genes implicated in microcephaly, CDK5RAP2, is also a homolog of PDE4DIP but lacks a DUF1220 domain (Cox et al., 2006). While these observations place the ancestral form of DUF1220 at the centrosome, and are consistent with the possibility that DUF1220 may be involved in centrosomal functions related to brain size, these findings should be viewed as suggestive and additional follow up investigations are needed.

In mice, the earliest progenitor cells (neuroepithelial cells) have an attachment to the apical surface. Early divisions bisect this attachment such that each daughter cell receives apical attachment proteins, remains attached to the apical surface, and (possibly because of this attachment) retains the neuroepithelial identity. This division is called a symmetrical proliferative division. Later in development, asymmetric divisions leave only one daughter cell attached to the apical surface. The cell that is no longer attached will migrate basally and become either a basal progenitor or a neuron. The number of rounds of symmetric cell divisions that occur before cells begin to divide asymmetrically is therefore a determinant in the final number of neurons, and this timing of when this switch occurs can have extreme effects on neuron number. Because DUF1220 expression in brain is restricted to neurons (Popesco et al., 2006), this may also explain why these scaling rules do not appear to apply to glial cells, even though they are derived from the same progenitor population. For example, it has been pointed out that 10 rounds of asymmetric cell division by one progenitor would produce 10 neurons. However, if all but the last round of cell division are symmetric, one progenitor will produce 512 neurons (Thornton and Woods, 2009). The type of cell division (symmetric vs. asymmetric) is thought to be controlled by the orientation of the centrosomes within the cell. The association between DUF1220 and brain size therefore suggests that the PDE4DIP, which is expressed at the centrosome and contains a DUF1220 domain, may play a role in this orientation.

### The primary cilia as a balance between proliferation and cell density-dependent differentiation

Primary cilia are sensory structures long thought to be the balance between further progression through the cell cycle and cell cycle exit. An effect on this structure might therefore be an ideal place to both sense local cell density and drive additional rounds of proliferation.

Vandepoele et al. showed that the *NBPF1* protein, which has seven DUF1220 domains, directly interacts with a protein called chibby (Vandepoele et al., 2010). Chibby is known to be involved in two cellular processes. The first is as a negative regulator of the Wnt/beta catenin pathway, wherein chibby is capable of migrating into the nucleus, binding beta catenin, and escorting it out of the nucleus, thereby preventing beta catenin-mediated transcription from occurring (Takemaru et al., 2003; Li et al., 2008). The second function ascribed to chibby occurs at the centriole, where it acts as a scaffold upon which the primary cilium is built. When a TOPFLASH assay was performed to investigate whether addition of *NBPF1* had any effect on Wnt/beta catenin signaling in DLD1 cells in culture, no change between *NBPF1* and mock induced cells was found. It is therefore possible that the nature of the *NBPF1*/chibby interaction is to influence primary cilia generation, which could affect the cell cycle. This would be consistent with the discovery that many *NBPF* genes contain an *EVI5* promoter that was duplicated from the unrelated *EVI5* gene that is known to encode a centrosomal protein (Vandepoele et al., 2009).

Primary cilia are well conserved structures, present in almost every mammalian cell type (Anderson et al., 2008). They are non-motile, sensory organelles, built during G1 of the cell cycle and mark the exit from the cell cycle, into G0. Conversely, resorption of the primary cilia is associated with reentry into the cell cycle (Plotnikova et al., 2009). This may be true because the centrioles nucleate both the primary cilium and the centrosome, which is necessary for mitosis. In this model, the centriole therefore functions as a fulcrum upon which exit from the cell cycle is determined.

Chibby is known to localize to the distal end of the mother centriole, upon which a primary cilium is built, and it is essential for primary cilium construction (Steere et al., 2012). It follows then that a protein capable of binding to chibby and preventing its use as a structural support during primary cilia generation would prevent the cell from exiting the cell cycle, and therefore force extra rounds of cell division. The known DUF1220 interaction with chibby therefore provides a plausible means by which extra rounds of neuronal cell divisions could be accomplished.

Circumstantial support can be found in many ependymomas, which are tumors of the ependymal cells that line the ventricles of the brain in adults (the cells that radial glia ultimately differentiate into). Interestingly, the two most common genetic aberrations found in these diseases are a gain of chromosome 1q (where most of the DUF1220 domains map) (Mendrzyk et al., 2006) or a loss of chibby (Karakoula et al., 2008).

### Neoteny

Heterochrony is any difference between a species and its ancestral form in the timing or duration of developmental events, such as neural progenitor proliferation. Neoteny occurs when the developmental window in the extant species is lengthened. In the absence of available data regarding rates of development in ancestral forms, extant species are frequently used instead. Indeed, it has been observed that the window of brain development has been extended from mouse to macaque, and from macaque to human (Kornack and Rakic, 1998), and this pattern is thought to extend to many other primate species (see http://bioinformatics.ualr.edu/ttime/home.php). In a primate model of brain development that emphasizes neuron number per unit time, rather than accumulation of cell mass, such a protracted window of neural development makes intuitive sense: more time spent in this stage may generate more neurons, and thus a bigger brain. Drawing out the window of development during which neural progenitor cells are generated, therefore, is a plausible mechanism by which DUF1220 may be influencing neural development.

Also it is worth mentioning that DUF1220 is expressed in several human tissues in addition to brain (Popesco et al., 2006), and there are several organs along with brain that exhibit neotenous developmental patterns in human.

A second, less obvious possibility is that the extra time humans spend in the window of neural development preserves the long distance connectivity important for higher cognitive function and changes in this timing may underlie some forms of cognitive disease. The human brain develops in an “inside out” pattern, with each wave of dividing neurons migrating past their predecessors and taking up residence basally to those cells. For normal brain connectivity, each wave most likely requires a certain amount of time to migrate to the appropriate location in the cortical plate and project its neurites outward. If cell division occurs too quickly and the next wave of dividing neurons is immediately behind the former, the developing cortex will get crowded very quickly. These cells will most likely not have enough time to send out neurites before being physically blocked by the next wave of neurons settling on top of them—particularly long distance projection axons.

This inability to form long distance projection neurons may therefore lead to an overly localized connectivity pattern, a brain related condition that has been associated with autism (Corchesne et al., 2007). The combination of not only how many neurons are made, but also how fast they are made, may therefore explain why greater than average brain growth in early postnatal years is associated with autism, and offers a potential explanation of why DUF1220 copy number increase can be associated with both macrocephaly (Dumas et al., 2012) and autism (Davis et al., 2014).

## Alternative models

With the increasing availability of primate genome sequences, the identification of genes, and other genomic variations that show HLS changes has accelerated (for a recent review see O'Bleness et al., 2012b), and a number of these have been potentially linked to human brain evolution.

### Microcephaly genes

As mentioned previously (section DUF1220 and the Centrosomal Effect on Brain Size), several genes have been identified that when mutated lead to autosomal recessive primary microcephaly including ASPM, microcephalin (MCPH), and CDK5RAP2 (Megraw et al., 2011). It is noteworthy that these all appear to encode proteins that are found at the centrosome, a feature has led to the suggestion that brain size may be influenced by genes that control centrosomal function. While some studies have shown that the ASPM gene coding region is under positive selection, this observation was not confirmed by a subsequent independent investigation and thus there remains some uncertainty about how involved these genes are in brain evolution.

### HAR1F

The human accelerated region 1F (HAR1F) gene was identified from a genome-wide search for genes that had undergone the most extreme human-specific sequence changes (Pollard et al., 2006). Though the precise function of HAR1F is unknown, it encodes an RNA product and is expressed at high levels in the fetal brain. While HAR1F shows a dramatic sequence divergence specifically in humans, the sequences do not change significantly in other mammalian (or primate) species, making it unlikely that it is responsible for primate brain expansion.

Additional work on human accelerated sequences has been carried out and a number of these may function as developmental enhancers (Capra et al., 2013). Also the NPAS3 gene, which is thought to encode a transcription factor, has been shown to contain a high number of human accelerated elements (Kamm et al., 2013a,b). Such rapidly evolving genomic sequences may have contributed to the evolution of human-specific traits and warrant further investigation.

### SRGAP2

The SRGAP2 gene, encoding a Slit-Robo GTPase, was reported to have undergone a human specific duplication that blocked the normal inhibitory domain of SRGAP2, resulting in an enhancement in its function (Dennis et al., 2012). Because SRGAP2 has been associated with neuronal migration, it has been suggested that this event may have produced an enhancement of this ability in the human brain. While it is possible that SRGAP2 may have contributed to recent human brain evolution, outside of humans it does not change in copy number to any appreciable degree (Dumas et al., 2012) and thus does not show the strong parallels between copy number increase and primate brain expansion as seen with DUF1220 domains.

However, it is worth mentioning that recent data has raised the possibility that some key brain-related gene changes may have been simultaneous. For example, the SRGAP2 duplication that has been proposed to have contributed to human brain evolution by effecting neuronal migration, is very near two *NBPF* (i.e., DUF1220 encoding) genes on chromosome 1 that also duplicated. Given DUF1220's role as a core duplicon that promotes duplication of other nearby sequences, it is possible that the SRGAP2 duplication was due to recombination events that were mediated by DUF1220 sequences. Should this be confirmed, it would imply that the SRGAP2 duplication and the duplication of the nearby *NBPF* genes, that added approximately 20 more copies of DUF1220 to the human genome, may have occurred as part of the same duplicative transposition event, i.e., at the same time and within the past 2 million years. It is plausible that such an instantaneous change, involving two potentially complementary brain processes (neuronal migration and neuron number), could have had a major sudden beneficial impact on human brain function.

### Gene expression

It has been suggested that changes in the regulation of genes, that alter their spatial and/or temporal expression, could be important to human evolution. While this view was based on minimal knowledge of the human and chimp genomes, the concept that gene regulation is important to evolutionary change remains a valid hypothesis and has been investigated using the latest genomic tools (Johnson et al., 2009). Also, modifications in enhancer sequences may be potential contributors to human brain evolution. The importance of such sequences in forelimb evolution has been reported, though not directly related to brain-related changes. Many other non-coding regions could in theory be contributors to the evolution of the primate brain, though testing of such candidate sequences is currently challenging.

Regarding mechanisms that could contribute to enhanced cognition, the data described earlier supports the view that the expansion of the primate brain, and the parallel increase in cognitive capacity, may be largely due to an increase in neuron number. While it is theoretically possible that changes in neurons, alone, are sufficient to lead to enhanced cognition, there may be other brain-related changes, e.g., involving glial cells, which also contribute. This possibility has been given recent support by the finding that transplanting human glia into a mouse brain is sufficient to lead to enhanced learning in a mouse (Han et al., 2013).

While the above genes and mechanisms show relevance to human brain size and/or evolution, none exhibit the striking changes across primate lineages that is seen with DUF1220 copy number and that parallels the increases in brain size found among these species. In addition, gene duplication (or domain duplication in the case of DUF1220) has long been recognized as a major mechanism underlying evolutionary change, and further that extremely duplicated sequences underlie extreme biological change (Rouquier et al., 2000). Given these observations, the fact that DUF1220 shows the largest number of HLS duplications (approximately 160 copies) of any protein coding region in the genome provides strong support that these sequences are major contributors to human-specific evolutionary change. In summary, we believe that after the above alternative theories and candidates are examined, none are comparable to the DUF1220 model in its ability to readily account for the progressive expansion of the primate brain as one moves from monkeys to apes to humans.

## Remaining questions

### Tissue and developmental expression

Immunocytochemistry studies of postmortem human brain indicate that, in adult human brain, DUF1220 protein expression is restricted to neurons (cell bodies and dendrites) and it is abundantly expressed in the neocortex, thought to be important in higher cognitive functions. However, DUF1220 protein is also expressed in several other human tissues (Popesco et al., 2006). If DUF1220 dosage is contributing to an increase in brain size, it is unclear what function it may have in other tissues in which it is also expressed. One possibility is that DUF1220 is involved in neoteny which as discussed earlier is thought to involve other developmental processes in addition to those related to brain If DUF1220 is having a proliferative effect that acts to enforce earlier cell division in the cell cycle at the expense of longer growth phases (therefore stimulating larger numbers of smaller cells), then one might expect the same trend seen in the primate brain to occur in other tissues in which DUF1220 is expressed, i.e., smaller cells but in greater numbers. Data for other organs in which DUF1220 is expressed does not yet exist to rigorously evaluate this hypothesis.

Also if DUF1220 is functioning to increase brain neuron number it can be expected that it will be expressed at developmental stages during which brain neurogenesis is particularly active. Investigation of this possibility is currently underway (see Author Note).

### DUF1220 domain proteins: structure and function

There are approximately 23 different DUF1220-encoding genes (*NBPF*) and they are predicted to encode a wide spectrum of different sized protein products. However, this is not the case and the primary HLS DUF1220-encoding protein that is detected in brain and other tissues is a single product of approximately 37 kDa. This may indicate that DUF1220 domains are processed (either post-transcriptionally or post-translationally) or possibly translated from unique internal initiation sites, in such a way that a 37 kDA protein product is consistently produced. Whether this product represents a specific form (e.g., an active or inactive form) or the only form is currently unknown. How this single protein product is generated and what function(s) it performs are topics currently under investigation.

### Benefits and limitations of modeling DUF1220 function in animal models

The use of transgenic mouse models to study gene function *in vivo* has had widespread utility, and these studies are currently underway in DUF1220 transgenic mice. However, while mice have one copy of DUF1220 in their genome, it is only the ancestral form (i.e., found in the PDE4DIP gene); they do not have the form that has increased in primates (i.e., the type encoded by the *NBPF* family). The primate form of DUF1220 was generated when the ancestral DUF1220 domain underwent a duplicative transposition, in which the DUF1220 copy in the PDE4DIP gene duplicated and the new copy inserted into a completely different genomic environment (O'Bleness et al., 2012a). This then became the NBPF-type DUF1220 family that greatly expanded in primates and has diverged considerably from the ancestral form. Mice have no *NBPF* genes and therefore no copies of DUF1220 outside of the one that is part of the PDE4DIP gene. While it is certainly possible that human DUF1220 copies will function normally when placed in the mouse genome, it is also possible that they may not function as they would when in the human (or any other primate) genome because the appropriate genomic environment is lacking in mouse. While these concerns are specific to “humanized” DUF1220 mice, an alternative approach is to remove the single DUF1220 domain in the mouse genome. Such DUF1220 KO mice have been generated, are viable and are currently under study. These represent the first animal model of DUF1220 function and should provide insight into the role of the ancestral DUF1220 domain.

## Conclusions

All the above data taken together point to a hypothesis regarding the evolutionary expansion of the anthropoid brain: Dosage of DUF1220 protein domains underlies the unique neuronal scaling rules seen in primates. Specifically, increases in DUF1220 dosage cause an increase in neuron number, resulting in a larger brain and greater cognitive ability. Whether the relatively consistent maintenance of cell size observed uniquely in primates is caused by DUF1220, and this is driving progenitor cell division, or if rapid cell division is causing neural cells to be smaller by spending less time in growth phases is not clear. Alternatively, it is also possible that neuronal cell size and density are not being held constant by DUF1220, but are simply not actively being modified by whatever other process affects brain size in non-primate mammals.

In summary, the remarkable increase in the copy number of sequences encoding DUF1220 protein domains in anthropoid species and particularly human, suggests that there were strong evolutionary selection pressures at work driving this expansion. Here we present data that suggests that the benefits accrued to the anthropoid lineages by virtue of increasing DUF1220 dosage were primarily related to brain evolution and cognitive capacity. Validation of this testable hypothesis awaits further investigation into the function of DUF1220 domains. In this regard, the model does make a number of testable predictions including (1) the unique brain evolution mechanism that appears to be unique to primates will be found to be manifest in the anthropoid lineages and will not evident in prosimians, (2) DUF1220 copy number will be linked to cognitive aptitude measures in the human population, and (3) DUF1220 will be shown to influence neuron number and/or processes that involve brain development and expansion among anthropoid primates.

While this hypothesis proposes that DUF1220 dosage is a key driver of primate brain evolution, it appears to have come at a very high cost. As mentioned earlier, DUF1220 sequences have undergone an unusually rapid and extreme increase in copy number in the 1q21 region. However this process has unfortunately also produced a highly disease-prone genome architecture for that region. For example, to date there have been at least a dozen different human diseases, including autism and schizophrenia, that have been linked to 1q21-associated CNVs (Dumas et al., 2012). In other words, many individuals receive highly deleterious versions of the 1q21 region of our genome as a result of the disease-prone, though evolutionarily adaptive, genome architecture of this region which has been largely shaped by DUF1220-related events. When viewed in this manner, this is the severe price that has been and continues to be paid for the large number of DUF1220 copies we have, and for our unusually large brain size and neuron number and exceptional cognitive capacity (Dumas and Sikela, 2009). One may hope that appreciation for how this process works will lead to much greater compassion for those who, through no choice of their own, are forced to carry this severe disease burden, while we fortunate ones are the beneficiaries of this same evolutionary process.

## Author note

Since this work was completed and submitted two reports appeared that provide additional support for the hypotheses presented here. First, transfection of DUF1220 sequences has been shown to promote proliferation of human neural stem cells (H9-derived) providing direct evidence in support of the view that DUF1220 may function by increasing neuron number (Keeney et al., in press). In addition, *in situ* hybridization analyses across human fetal brain development, shows that DUF1220 domains are expressed in the ventricular zone and primarily during human cortical neurogenesis, and therefore are expressed at the right time and place to be affecting cortical brain development. Second, it has been demonstrated that dolphins, a non-primate mammal with a relatively large brain and high cognitive capacity but low DUF1220 copy number, show unusually strong selection on the protein coding regions of genes previously implicated in human brain function and psychiatric disorders, while other species including humans do not show such coding region selection (Ogawa and Vallender, 2014). These results suggest that dolphin (cetacean) brain evolution may have taken a path distinct from that utilized in the primate order and are consistent with the hypothesis that primate brain evolution may have primarily utilized a unique genetic mechanism, DUF1220 dosage increase, to facilitate brain expansion.

### Conflict of interest statement

James M. Sikela is founder and shareholder of GATC Science, LLC a biotech company focused on genomics. The authors declare that the research was conducted in the absence of any commercial or financial relationships that could be construed as a potential conflict of interest
